# Supplementary material for: The U3 and Env Proteins of Jaagsiekte Sheep Retrovirus and Enzootic Nasal Tumor Virus Both Contribute to Tissue Tropism
Source: Viruses. 2019 Nov 14;11(11):1061. doi: 10.3390/v11111061 (PMC6893448; doi:10.3390/v11111061)

Supplementary Table 1.

	Primer sequences used to produce megaprimer	
**Chimera (Chm)**	**Forward (FWD) Primer Sequences**	**Reverse (REV) Primer Sequences**
**1**	5’- gaataaacaagttatgtactttataaatatagcattgtaataaagcaaggtatcagccattcttggtctg -3’	5’- acggagcgtcctcgctaagaaaataagagagagaccgcagccagcacggacaaaag -3’
**2**	5’- aggaggagtagtaaggtatatagttgagagtataaatatggggcaaacacatagtcgtcaattg-3’	5’- acccagtttcccgaaaccgggggtaaaggattaccttgaacatctgttttagaccggcaatc-3’
**3**	**Megaprimer 1**	
	5’- catgtttgtgtttttccacagaatgccgaagcaccgcgctggatc -3’	5’- gctcttaagacttcctgagggtggcgggaca -3’
	**Megaprimer 2**	
	5’- atgcaacgcatgacgctgagcgagcccacgagtgacatgtttgtgtttttccacagaatg-3’	5’-agcagtgccaaaagcaaacatccgagccttaagagctcttaagacttcctgagggtggcgggaca -3’
**4**	5’- gagatgcgggggacgacccgtgagggggacaacccgcggagggttaa -3’	5’- tgagaggatcagaccaaaatggctgatactctgctttattgtgcagcagtat-3’
**5**	Chm4 FWD	5’- gttgagaggatcagaccaagaatggctgataccttgctttattgtgcagcagtat-3’
**6**	**Megaprimer 1**	
	Chm3 FWD 1	Chm4 REV
	**Megaprimer 2**	
	Chm3 FWD 2	Chm4 REV

Supplementary Material S1.

A)

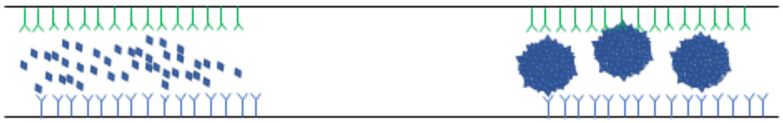

B)

Virus	p27 (pg/mL)	IFU/mL
**JSRV**	$5.7 \times 10^3 \pm 1.3 \times 10^{3\S}$	$4.9 \times 10^7 \pm 1.8 \times 10^{7\S}$
**ENTV**	$3.4 \times 10^3 \pm 5.5 \times 10^2$	$2.3 \times 10^7 \pm 9.9 \times 10^6$
**Chm1 – ERU5**	$8.0 \times 10^3 \pm 1.6 \times 10^3$	$6.9 \times 10^7 \pm 2.4 \times 10^7$
**Chm2 - E*gag***	$6.2 \times 10^3 \pm 4.6 \times 10^2$	$5.3 \times 10^7 \pm 1.7 \times 10^7$
**Chm3 - E*env***	$1.3 \times 10^3 \pm 4.2 \times 10^2$	$1.1 \times 10^7 \pm 4.5 \times 10^6$
**Chm4 – EU3**	$1.6 \times 10^3 \pm 1.4 \times 10^2$	$1.3 \times 10^7 \pm 4.3 \times 10^6$
**Chm5 - ELTR**	$5.3 \times 10^3 \pm 1.1 \times 10^3$	$4.5 \times 10^7 \pm 1.6 \times 10^7$
**Chm6 – E*env*U3**	$3.1 \times 10^3 \pm 4.9 \times 10^2$	$2.7 \times 10^7 \pm 9.1 \times 10^6$

§mean ± SEM

Supplementary Material S2.

Protocol Day	Procedure	Description
Day 0	Control serum collection	Pre-immunization bleed (0.1 mL per mouse)
Day 1	First injection	Immunize with 25 µg antigen IM* in 4 flanks
Day 21	First booster	Boost with 10 µg antigen IM in 2 flanks
Day 42	2nd booster	Boost with 10 µg antigen IM in 2 flanks
Day 50	Test-bleed	Test-bleed (0.1 mL per mouse); p27 ELISA
Day 62	3rd booster	Boost with 10 µg antigen IM in 2 flanks
Day 70	Test-bleed	Test-bleed (0.1 mL per mouse); p27 ELISA
Day 75	End point	Terminal bleed (heart puncture, yielding 0.5–1 mL per mouse); p27 ELISA

*IM stands for intramuscular

Supplementary Material S3.

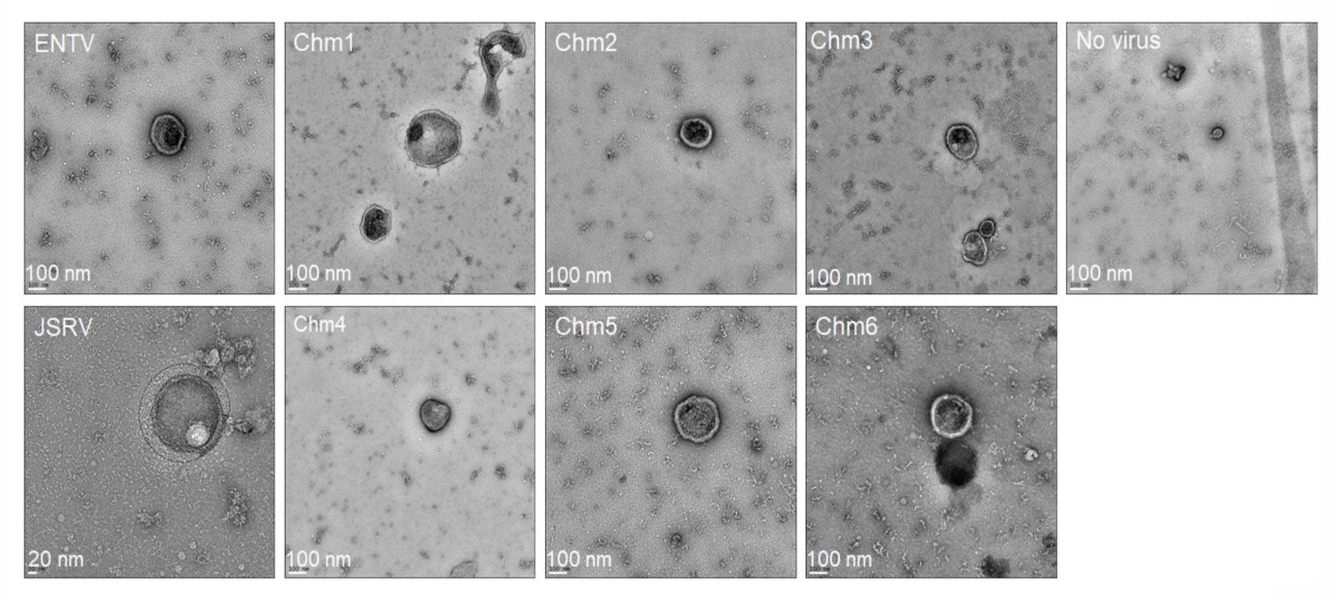

Supplementary Materia S4.

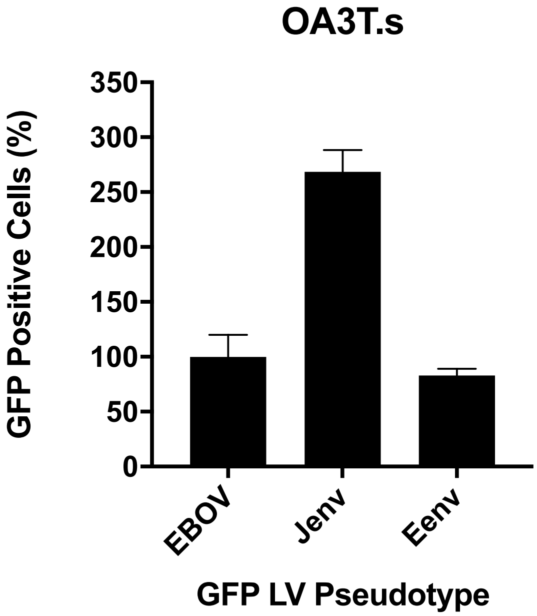

Supplement: Supplementary file 1 [file viruses-11-01061-s001.pdf]
